# Elevation of astrocyte-derived extracellular vesicles over the first month post-stroke in humans

**DOI:** 10.1038/s41598-024-55983-w

**Published:** 2024-03-04

**Authors:** Matthew A. Edwardson, Masato Mitsuhashi, Dennis Van Epps

**Affiliations:** 1 Department of Neurology, Georgetown University, Washington, DC USA; 2 Research Division, MedStar National Rehabilitation Hospital, Washington, DC USA; 3 NanoSomiX, Inc., Irvine, CA USA

**Keywords:** Ischemic stroke, Extracellular vesicles, Exosomes, Hemorrhagic stroke, Astrocytes, Neural cell adhesion molecule L1, Excitatory amino acid transporter 1, Myelin oligodendrocyte glycoprotein, Stroke, Prognostic markers

## Abstract

We sought to identify alterations in the quantity of plasma brain-derived extracellular vesicles (EV) over the first month post-stroke to shed light on related injury and repair mechanisms. We assessed plasma levels of presumed neuron-derived EVs (NDEs), astrocyte-derived EVs (ADEs), and oligodendrocyte-derived EVs (ODEs) in 58 patients 5, 15, and 30 days post-ischemic stroke and 46 controls matched for cardiovascular risk factors using sandwich immunoassays. Subsets of brain-derived EVs were identified by co-expression of the general EV marker CD9 and markers for neurons (L1CAM, CD171), astrocytes (EAAT1), and oligodendrocytes (MOG) respectively. Clinical MRIs assessed lesion volume and presence of hemorrhagic transformation. ADE levels were elevated 5, 15, and 30 days post-stroke compared to controls (*p* = 0.002, *p* = 0.002, and *p* = 0.005 respectively) with no significant change for NDE or ODE. ADEs were increased 15 days post-stroke in patients with hemorrhagic transformation (*p* = 0.04) compared to patients with no hemorrhage. We conclude that ADE levels are preferentially increased over the first month post-stroke in humans, possibly to provide trophic support to injured neurons following ischemia. ADEs hold potential as biomarkers of blood–brain barrier breakdown and hemorrhagic transformation, but this requires further study at earlier time points post-stroke.

## Introduction

Extracellular vesicles (EVs) are small vesicles (< 150–1000 nm) with a lipid bilayer produced by nearly all cell types^1^. EVs contain proteins, lipids, and RNAs^1^. Their primary role was once thought to be cellular waste removal, but EVs are increasingly implicated in intercellular communication^2,3^. EVs can cross the blood–brain barrier^4^. Investigators have isolated EVs of presumed neural, astrocytic or oligodendrocytic origin using immunoassays for their respective cell surface proteins^5^. This has sparked great interest in isolating brain-derived EVs (BDEs) from the peripheral blood to characterize physiologic changes in the CNS that are otherwise inaccessible in living humans.

BDEs are increasingly reported as biomarkers in neurological disease, but few have studied them in clinical stroke. Plasma neuron-derived EVs (NDEs) were shown to contain increased levels of pathologic proteins in Alzheimer’s patients compared to controls and in some cases even predict progression to dementia in healthy elderly adults^6–8^. Plasma astrocyte-derived EVs (ADEs) were decreased after traumatic brain injury, but contained dramatically increased complement levels compared to controls^9^. In ischemic stroke, total blood EV levels increase acutely and may promote inflammation^10,11^. Looking more specifically at BDEs, only one study was identified related to clinical stroke^12^. The BAPTISe investigators found that low NDE levels around 5–45 days after stroke were associated with worse outcomes on the Barthel Index at 6-months^12^. Preclinically, most studies suggest BDEs play a role in neural repair after ischemic injury^13–16^.

BDEs may also serve as biomarkers of damage to the blood–brain barrier (BBB) after stroke. From a theoretical perspective, one would expect small vesicles to more easily pass from the CNS to the peripheral blood in the setting of BBB breakdown. There are 2 main peaks of BBB permeability after stroke, the first around 6–12 h related to acute hypoxic injury and the second at 2–4 days from neuroinflammation^17,18^. From 1 week to 1 month post-stroke increased permeability continues due to small leaky blood vessels created in the process of angiogenesis^18^. Most hemorrhagic transformation of ischemic stroke takes place during the second peak of BBB permeability 2–4 days after stroke, and occurs in around 9% of patients^19^. Patients with hemorrhagic transformation have significantly greater morbidity and mortality^19,20^. As it pertains to BDEs, a prior study showed increased NDE levels immediately after aortic arch replacement surgery that subsided over time and correlated with post-operative delirium^21^. No prior studies have directly assessed multiple BDE types (neural, astrocytic, oligodendrocytic) following human ischemic stroke, nor have they assessed BDEs in the setting of hemorrhagic transformation.

In the current study we sought to assess levels of double-stained vesicles (CD171 + CD9 +, EAAT1 + CD9 +, and MOG + CD9 +), hereafter referred to as NDEs, ADEs, and oligodendrocyte-derived EVs (ODEs) respectively, over the first month post-stroke. The period from 5 to 30 days after ischemic stroke represents an important transition from CNS injury to repair. We hypothesized that BDE levels would evolve over time, with primarily NDEs and ADEs elevated early after ischemia, and ODEs elevated late during the process of remyelination. We further hypothesized that all BDE types would be elevated early (5 days post-stroke) in patients who experienced hemorrhagic transformation compared to those without hemorrhage.

## Results

### Participant characteristics

Stroke participants had moderate to severe disability at day 5 (mean modified Rankin Scale = 4.3 ± 0.7) that improved over the course of 30 days (Table 1). The stroke and control groups were well-matched with regard to age, sex, race, ethnicity, and cardiovascular comorbidities with the exception of hyperlipidemia (Table 2). Twenty-eight percent of stroke participants received TPA and 12% received thrombectomy. The TOAST classifications were as follows: 34% lacunar, 24% cardioembolic, 19% undetermined etiology, 17% large artery atherosclerosis, and 5% other determined etiology.

**Table 1:** Blood sample collection timing and functional measures for ischemic stroke patients.

	5-day (n = 58)	15-day (n = 58)	30-day (n = 58)
Time from stroke onset to study visit (days)	5 (1.7)	15.5 (2.5)	33.1 (6.5)
mRS (0–6)	4.3 (0.7)	3.9 (0.9)	3.6 (0.9)
NIHSS (0–42)	8.3 (3.5)	6.7 (3.8)	5.9 (3.9)
UE-FM (0–66)	15.5 (13.3)	20.4 (17.8)	26.2 (19.8)

Data are mean (SD).

*mRS* modified Rankin Scale, *NIHSS* NIH stroke scale, *UE-FM* upper extremity Fugl-Meyer.

**Table 2:** Baseline characteristics of ischemic stroke patients and matched controls, including standardized differences to gauge match fidelity.

	Ischemic Stroke (n = 58)	Control (n = 46)	Standardized Difference*
Age	65.9 (11.2)	66.3 (10.6)	0.04
Sex			
Male	32 (55%)	24 (52%)	0.06
Female	26 (45%)	22 (48%)	
Race			
African American	35 (60%)	26 (56%)	0.08
White	20 (35%)	17 (37%)	0.05
Asian	3 (5%)	3 (7%)	0.06
Ethnicity			
Non-Hispanic	57 (98%)	45 (98%)	0.03
Hispanic	1 (2%)	1 (2%)	
Cardiovascular comorbidities			
Hypertension	48 (83%)	37 (80%)	0.06
Diabetes	23 (40%)	17 (37%)	0.06
Hyperlipidemia**	26 (45%)	39 (85%)	0.92
Atrial fibrillation	6 (10%)	3 (7%)	0.14
Current smoker	14 (24%)	10 (22%)	0.06
On statin at time of blood draw	56 (97%)	42 (91%)	0.22
Hemorrhagic transformation			
Yes	12 (21%)		
No	42 (79%)		
Received tissue plasminogen activator (tPA)			
Yes	16 (28%)		
No	42 (72%)		
Received thrombectomy			
Yes	7 (12%)		
No	51 (88%)		
TOAST classification			
Large artery atherosclerosis	10 (17%)		
Cardioembolic	14 (24%)		
Lacunar	20 (34%)		
Other determined etiology	3 (5%)		

Data are mean (SD) or n (%).

*The ideal standardized difference is < 0.1^22^; for sample size = 50 with a dichotomous variable equally distributed in the population a standardized difference = 0.1 equates to a *p* value = 0.62.

**The variables statin use and hyperlipidemia interact. We prioritized matching for statin use over hyperlipidemia during control recruitment.

### Extracellular vesicle levels in ischemic stroke participants

**Figure 1:**
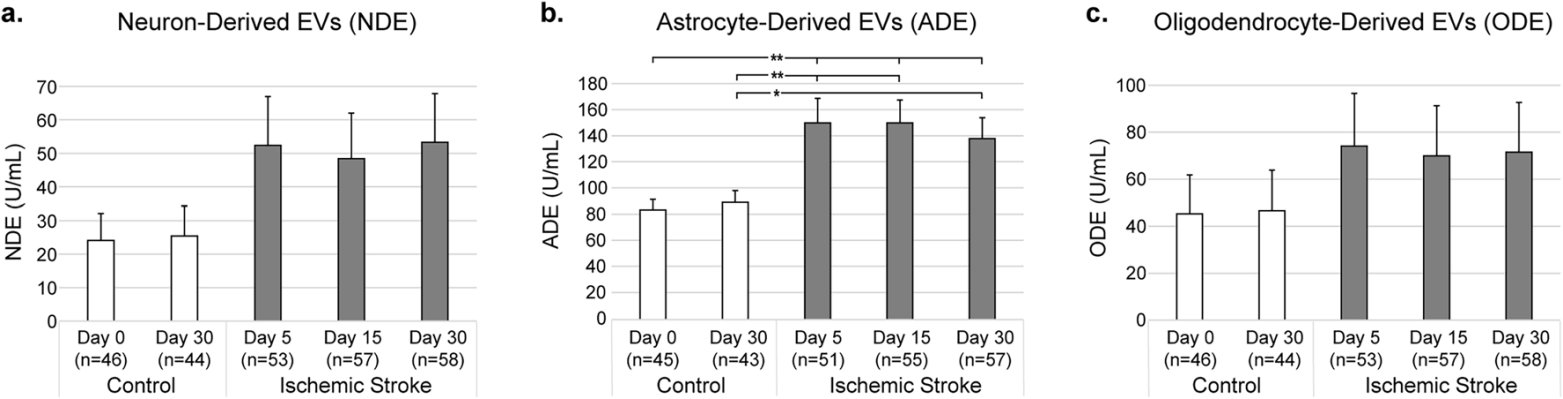
Extracellular vesicle (EV) concentrations (mean ± SE) in ischemic stroke versus control participants for (**a**) neuron-derived, (**b**) astrocyte-derived, and (**c**) oligodendrocyte-derived EVs (mean ± SE). There were no significant differences between the controls from day 0 to day 30. Wherever n < 46 for control or n < 58 for ischemic stroke there were samples that returned no measurable result. *p < 0.05; **p < 0.01.

In order to determine whether stroke affected the levels of NDEs, ADEs and ODEs, the quantities of these vesicles in the stroke (n = 58) and control (n = 46) populations (Fig. 1) were compared. A small number of the samples were either missing or the assay returned no measurable result, so these were treated as missing data. We found that the level of ADEs were significantly elevated in stroke participants compared to controls 5, 15, and 30 days post-stroke (mean ± SE at 5 days: 149.9 ± 18.8 U/mL vs. 83.6 ± 7.5 U/mL, *p* = 0.002; 15 days: 149.8 ± 17.7 U/mL vs. 83.6 ± 7.5 U/mL, *p* = 0.002; 30 days: 138 ± 15.9 U/mL vs. 83.6 ± 7.5 U/mL, *p* = 0.005). The increase for the NDEs and ODEs did not reach the level of significance (NDE vs. control at 5, 15, and 30 days respectively: *p* = 0.11, *p* = 0.15, *p* = 0.11; ODE vs. control at 5, 15, and 30 days respectively: *p* = 0.32, *p* = 0.38, and *p* = 0.35). The levels of NDE, ADE and ODE were also resampled in the control population 30 days after the original samples were drawn and no differences were found over time for each of the 3 EV types (NDE: *p* = 0.91; ADE: *p* = 0.62; ODE: *p* = 0.95). In summary, ADEs were the only vesicle type significantly elevated 5–30 days post-stroke and there were no significant changes over time among the controls.

### Extracellular vesicle levels and lesion size

**Figure 2:**
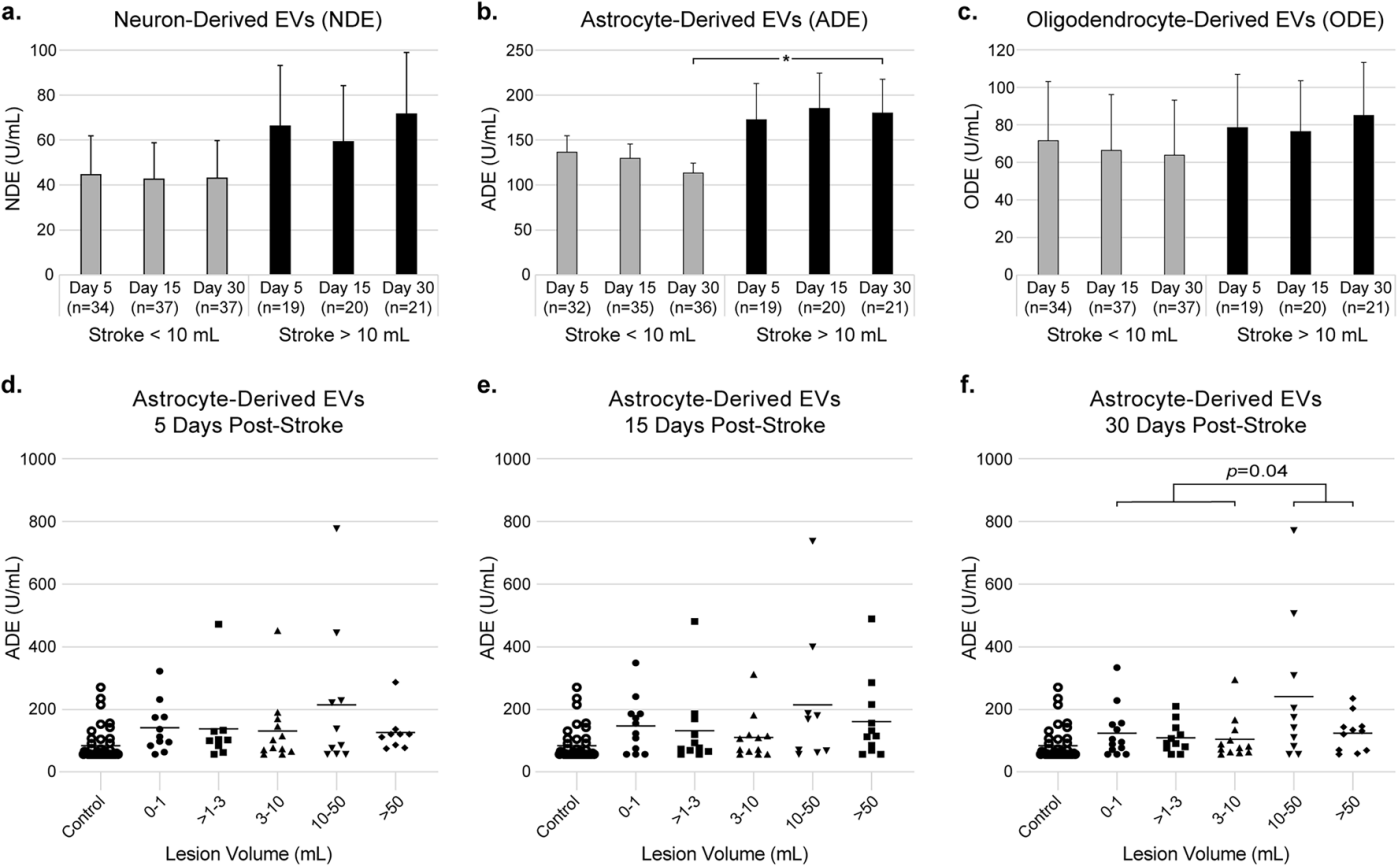
Stroke volume < 10 mL versus > 10 mL in ischemic stroke participants and their corresponding extracellular vesicle (EV) concentrations (mean ± SE) 5, 15, and 30 days post-stroke for (**a**) neuron-derived, (**b**) astrocyte-derived, and (**c**) oligodendrocyte-derived EVs. Wherever n < 37 for volume < 10 mL or n < 21 for volume > 10 mL there were samples that returned no measurable result. Astrocyte-derived EV concentrations for individual participants (bar represents mean) at (**d**) 5 days, (**e**) 15 days, and (**f**) 30 days post-stroke. *p < 0.05.

We next determined whether NDE, ADE, or ODE levels were associated with stroke lesion size. The mean stroke lesion size ± SE among all participants was 22.8 ± 5.1 mL (range 0.2–156.8 mL). We separated the participants into those with lesions > 10 mL (n = 21) and those with lesions < 10 mL (n = 37) thinking that lesions > 10 mL would reflect more significant brain injury and plotted NDE, ADE and ODE levels over time (Fig. 2). There was a significant increase in ADEs in those with lesions > 10 mL compared to those with lesions < 10 mL, but only at 30 days post-stroke (mean ± SE at 5 days: 172.5 ± 40.3 U/mL vs. 136.5 ± 18.2 U/mL, *p* = 0.36; 15 days: 185.2 ± 39.2 U/mL vs. 129.6 ± 16 U/mL, *p* = 0.13; 30 days: 180.1 ± 37.4 U/mL vs. 113.4 ± 11.2 U/mL, *p* = 0.04). The increase for the NDEs and ODEs for lesions > 10 mL vs. < 10 mL did not reach the level of significance (NDE at 5, 15, and 30 days respectively: *p* = 0.48, *p* = 0.56, *p* = 0.35; ODE at 5, 15, and 30 days respectively: *p* = 0.88, *p* = 0.82, and *p* = 0.64). Thus, there were only weak associations between the number of brain-derived EVs and lesion size.

### Extracellular vesicle levels and hemorrhagic transformation

**Figure 3:**
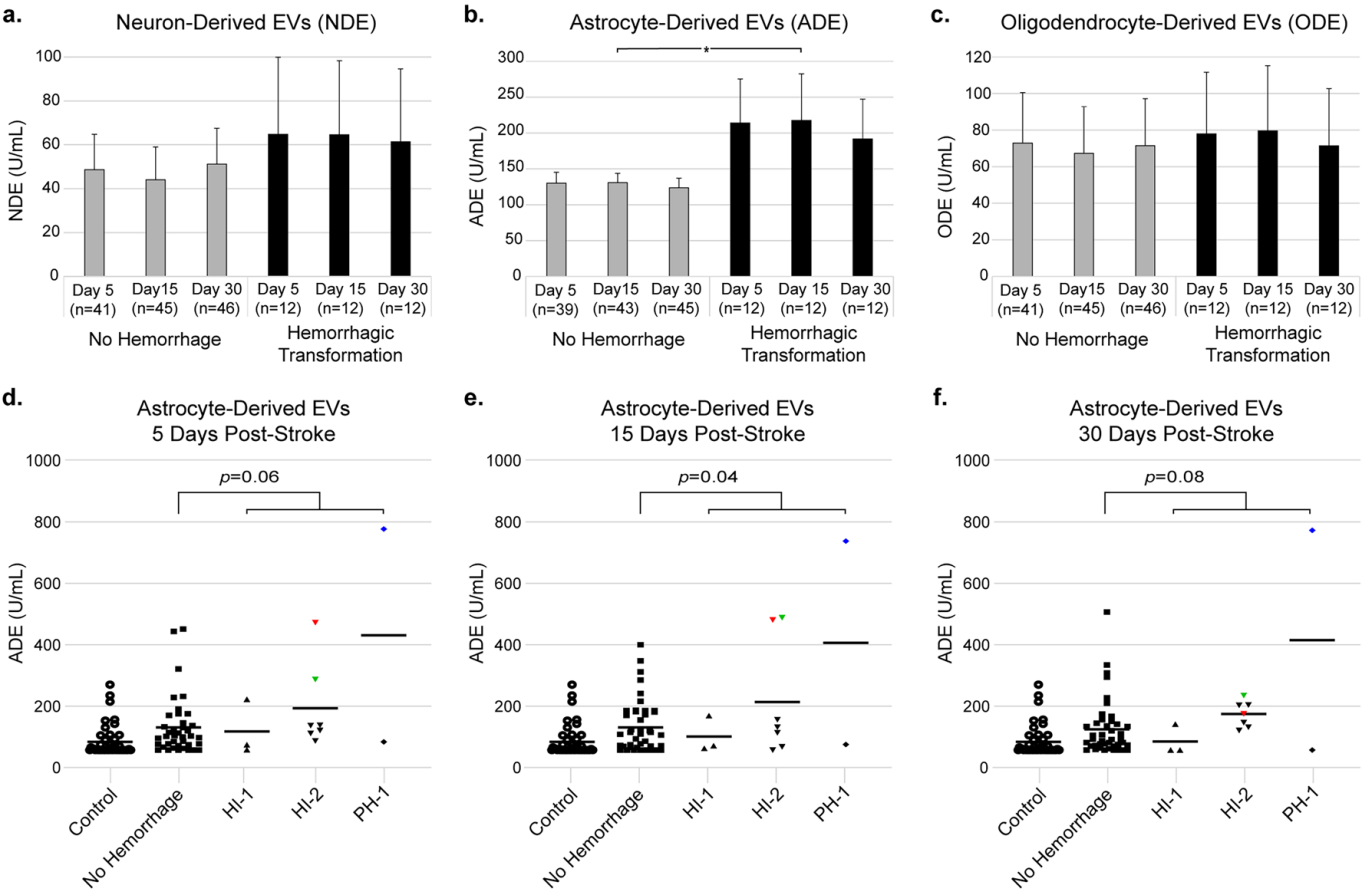
Hemorrhagic transformation status for ischemic stroke patients and their corresponding extracellular vesicle (EV) concentrations (mean ± SE) at 5, 15, and 30 days post-stroke for (**a**) neuron-derived, (**b**) astrocyte-derived, and (**c**) oligodendrocyte-derived EVs. Wherever n < 46 for no hemorrhage there were samples that returned no measurable result. Astrocyte-derived EV (ADE) concentrations for individual participants (bar represents mean) at (**d**) 5 days, (**e**) 15 days, and (**f**) 30 days post-stroke. The 3 outliers with the highest ADE levels 15 days post-stroke are labeled in red, green, and blue to show how these outliers fluctuated over time. *HI-1* hemorrhagic infarction type 1, *HI-2* hemorrhagic infarction type 2, *PH-1* parenchymal hematoma type 1 (listed in order of increasing severity). *p < 0.05.

We subsequently assessed whether the number of NDEs, ADEs or ODEs were associated with hemorrhagic transformation (Fig. 3). Twelve participants were confirmed to have hemorrhagic transformation on neuroimaging (3 with HI-1, 7 with HI-2, and 2 with PH-1). There was a significant increase in ADEs in those with hemorrhagic transformation at day 15 as well as trends toward an increase in ADEs at days 5 and 30 (mean ± SE at 5 days: 214 ± 61.4 U/mL vs. 130.2 ± 15.1 U/mL, *p* = 0.06; 15 days: 217.6 ± 64.7 U/mL vs. 130.9 ± 13 U/mL, *p* = 0.04; 30 days: 191.6 ± 55.5 U/mL vs. 123.7 ± 13.3 U/mL, *p* = 0.08). There were no significant differences in levels of NDEs or ODEs between participants with and without hemorrhagic transformation (NDE at 5, 15, and 30 days respectively: *p* = 0.65, *p* = 0.54, *p* = 0.78; ODE at 5, 15, and 30 days respectively: *p* = 0.93, *p* = 0.82, and *p* = 1.0). To summarize, hemorrhagic transformation was associated with an increase in ADE levels 15 days post-stroke and trends toward an increase at 5 and 30 days post-stroke, but was not associated with NDE and ODE levels.

### Relationship between lesion size and hemorrhagic transformation

**Figure 4:**
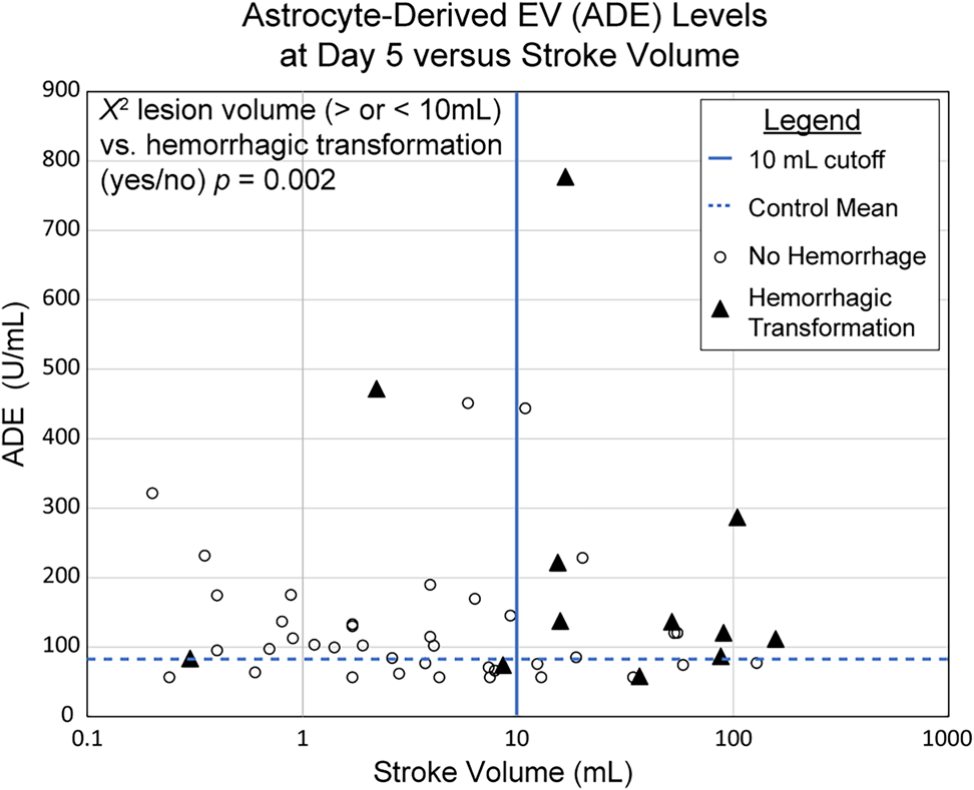
Astrocyte-derived extracellular vesicle concentrations 5 days post-stroke versus ischemic stroke lesion volume, including designation of which participants had hemorrhagic transformation (triangles) and those with no hemorrhage (circles). Stroke lesion volume and hemorrhagic transformation were closely related, as evidenced by a chi-squared test with *p* = 0.002.

The mean lesion size ± SE for those with and without hemorrhagic transformation was 48.8 ± 14.4 mL and 16 ± 4.8 mL respectively. A chi-squared test using lesion size greater or less than 10 mL suggested that lesion size and hemorrhagic transformation were significantly related (*X*^2^ (1,58) = 9.9, *p* = 0.002). A plot of ADE levels at 5 days post-stroke vs. stroke volume (Fig. 4) confirmed that lesion volume was more closely tied to hemorrhagic transformation than ADE levels.

### Extracellular vesicle levels and tPA, thrombectomy, and TOAST classification

**Figure 5:**
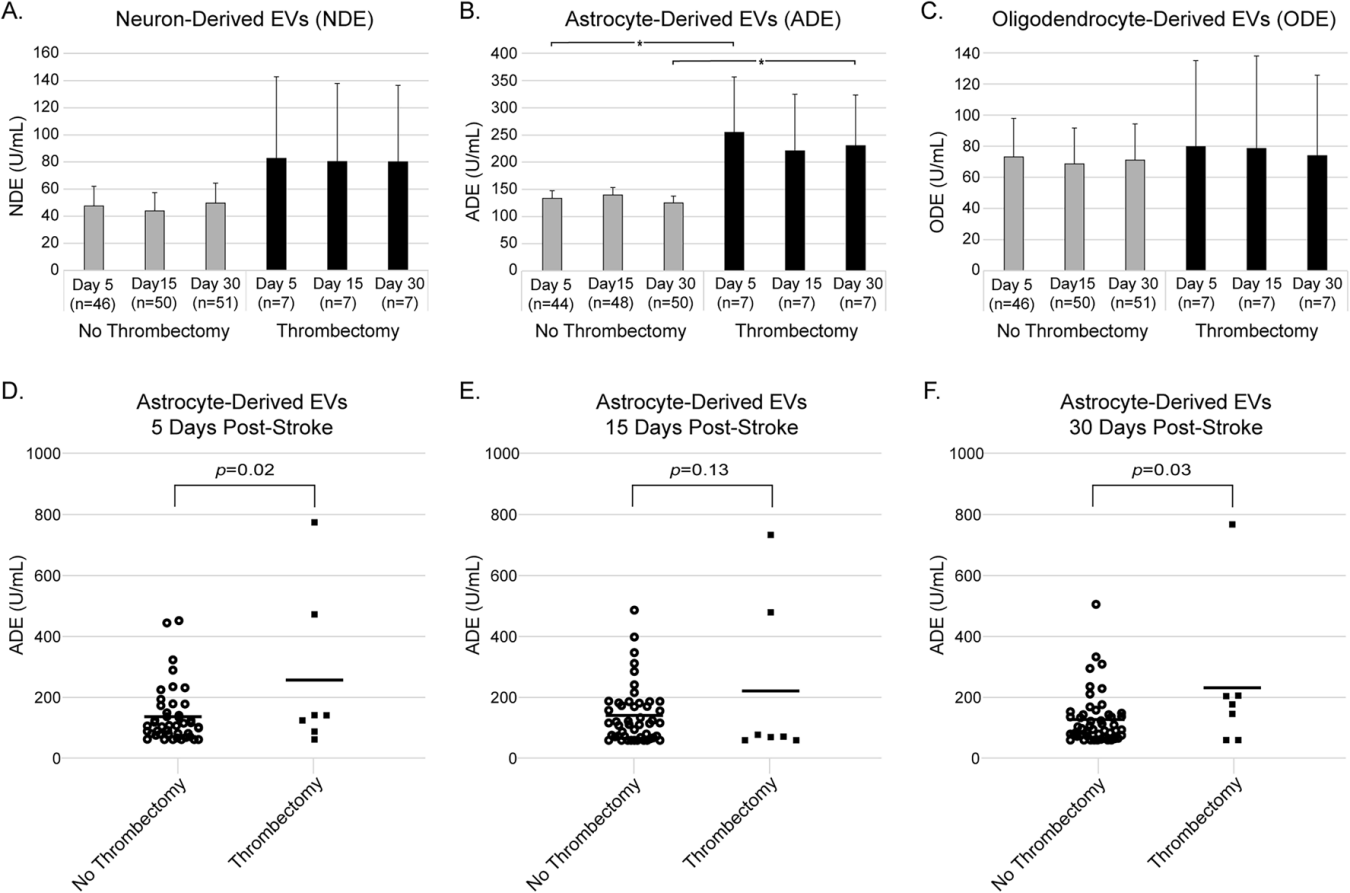
Mechanical thrombectomy status for ischemic stroke patients and their corresponding extracellular vesicle (EV) concentrations (mean ± SE) at 5, 15, and 30 days post-stroke for (**a**) neuron-derived, (**b**) astrocyte-derived, and (**c**) oligodendrocyte-derived EVs. Wherever n < 51 for no thrombectomy there were samples that returned no measurable result. Astrocyte-derived EV (ADE) concentrations for individual participants (bar represents mean) at (**d**) 5 days, (**e**) 15 days, and (**f**) 30 days post-stroke. *p < 0.05.

In our final analysis we sought to determine whether EV levels were dysregulated in patients receiving IV-tPA (Alteplase), mechanical thrombectomy, or with strokes of different etiologies. There were no significant differences in levels of NDEs, ADEs or ODEs between participants who did or did not receive IV-tPA (Suppl. Fig. S1; NDE at 5, 15, and 30 days respectively: *p* = 0.69, *p* = 0.74, *p* = 0.6; ADE at 5, 15, and 30 days respectively: *p* = 0.4, *p* = 0.91, *p* = 0.52; ODE at 5, 15, and 30 days respectively: *p* = 0.45, *p* = 0.47, and *p* = 0.39). There was a significant increase in ADEs in those who received mechanical thrombectomy at day 5 and 30 compared to those who did not receive thrombectomy (Fig. 5; mean ± SE at 5 days: 254.9 ± 101.7 U/mL vs. 133.2 ± 14.1 U/mL, *p* = 0.02; 15 days: 220.7 ± 101.7 U/mL vs. 139.5 ± 13.8 U/mL, *p* = 0.13; 30 days: 230.3 ± 93.3 U/mL vs. 125 ± 12.3 U/mL, *p* = 0.03). There were no significant differences in levels of NDEs or ODEs between participants who did or did not receive thrombectomy (NDE at 5, 15, and 30 days respectively: *p* = 0.42, *p* = 0.38, *p* = 0.5; ODE at 5, 15, and 30 days respectively: *p* = 0.92, *p* = 0.88, and *p* = 0.97). Five out of 7 patients who underwent thrombectomy had hemorrhagic conversion, 3 with HI-2 and 2 with PH-1. The mean lesion volume in participants receiving thrombectomy was 21.8 mL. There were no differences in EV levels between strokes of different etiologies according to the TOAST classification for each type of BDE at each respective time point (Suppl. Fig. S2; ANOVA NDE at 5, 15, and 30 days respectively: *p* = 0.93, *p* = 0.85, *p* = 0.93; ADE at 5, 15, and 30 days respectively: *p* = 0.46, *p* = 0.48, *p* = 0.31, ODE at 5, 15, and 30 days respectively: *p* = 0.99, *p* = 0.96, and *p* = 0.98). In summary, mechanical thrombectomy was associated with increased ADE levels at certain time points post-stroke which may be related to the high rate of hemorrhagic transformation in this population.

## Discussion

A principal finding from this study was that plasma astrocyte-derived extracellular vesicles (ADEs) remained significantly elevated 5–30 days post-ischemic stroke. We also found weak associations between ADE levels and stroke lesion size, hemorrhagic transformation, and mechanical thrombectomy. These findings could shed light on post-stroke physiology.

The most consistent finding in this study was that ADE levels remained significantly elevated from 5 to 30 days post-ischemic stroke. The neural- and oligodendrocyte-derived EV levels appeared slightly increased, but with this sample size were no different than matched controls statistically. One possible explanation could be that all brain cells produce more EVs after stroke and astrocytes simply outnumber other cell types in the human brain allowing us to find a statistically significant difference for ADEs in this study of modest sample size. A long held misconception in the field suggested a 10:1 ratio of glial to neural tissue in the human brain^23^, but this was later shown to be true only in white matter^24^. In addition, oligodendrocytes outnumber astrocytes throughout the brain in a 3:1 ratio^24^. Therefore, a more likely explanation for our findings is that stroke preferentially induces the release of ADEs over vesicles from other cell types. Indeed, ADEs were shown to increase by 34% in rodent brain tissue 24 h post-stroke whereas NDEs, ODEs, and microglial EVs remained unchanged^15^. Prior human studies demonstrated an increase in serum or plasma EVs post-stroke, but none looked specifically at ADEs^10,25^.

Astrocytes undergo hypertrophy with elongated processes within minutes of cerebral ischemia and form a glial scar within days, presumably to provide trophic support to injured neurons and prevent the spread of ischemic cell death^26,27^. Reactive astrogliosis may last for months to years depending on the severity of the injury^28^. This timeframe for astrocyte activation is in line with our finding of increased ADEs from 5 to 30 days post-stroke. Some have shown evidence that ADEs can provide neural protection or combat reactive oxygen species in neuronal cell lines subjected to hypoxic ischemic conditions^29,30^. Over the first month post-stroke the brain is likely transitioning from neural injury to repair. ADE secretion during this timeframe could be due to ongoing neurotrophic support to injured neurons or the promotion of neural repair. For example, one study found evidence that ADEs subjected to oxygen glucose deprivation and treated with a semaphorin 3A inhibitor promoted axonal outgrowth in ischemic neurons^16^.

We expected the concentration of all brain-derived EVs to increase in plasma in the setting of hemorrhagic transformation since hemorrhage is associated with breakdown of the blood–brain barrier, but only found an association with ADE populations. A simple explanation might be that because ischemia induces a preferential increase in ADEs they are more likely to pass through a damaged BBB. An alternative explanation could stem from the close proximity of astrocytes to endothelial cells in the neurovascular unit. Astrocytic end feet surround the endothelial cells and help maintain the integrity of the BBB^18^. In the setting of ischemia both astrocytes and endothelial cells have impaired integrin signaling, contributing to breakdown of the BBB^31^. It follows that ADEs would preferentially enter the plasma over other brain-derived EVs in the setting of BBB disruption.

Prior studies have shown, and we demonstrated here, that hemorrhagic transformation is closely tied to stroke lesion size^19,32^. Our findings suggest that lesion size is more closely related to hemorrhagic transformation than ADE levels. It remains possible, however, that brain-derived EVs levels in the hyperacute phase (< 24 h post-stroke) or specific proteins within EVs may prove to be stronger biomarkers of hemorrhagic transformation.

We also found increased ADE levels at 5 and 30 days post-stroke in patients who received mechanical thrombectomy. Five of the 7 patients who received thrombectomy had moderate levels of hemorrhagic transformation (HI-2 or PH-1). Bleeding into the infarct bed is common after thrombectomy, though most studies only report the rate of severe hemorrhagic conversion (PH-2) which was 5.1% in a pooled analysis of thrombectomy trials^33^. We suspect that increased ADE levels following mechanical thrombectomy were related to breakdown of the blood–brain barrier due to reperfusion of ischemic endothelial cells.

The findings in this study raise interesting questions about the pathophysiology of stroke and provide future avenues for research. The first question is why are ADEs increased over the first month post-stroke? Possibilities include the transfer of neurotrophins to ischemic neurons, recruitment of microglial cells or macrophages, and promotion of axonal outgrowth following ischemia. Systems biology approaches that identify the protein, RNA, or lipid content of ADEs may help narrow in on their role in human stroke physiology.

The other important question is whether ADEs are useful to identify patients at risk for hemorrhagic transformation. We found a weak association between an increase in ADEs and hemorrhagic transformation at specific time points post-stroke driven by 2–3 outliers with very high levels. A diagnostic test with a high cutoff for ruling in major risk of hemorrhagic transformation (test with high specificity) could be helpful to choose which patients to avoid treating with thrombolytics. The current study was limited in that we had no hyperacute (< 24 h) plasma samples. In addition, we had no patients who experienced the most severe degree of hemorrhagic transformation, PH-2, which leads to significant clinical deterioration and in some cases death. Such a diagnostic test would have to be accurate in the hyperacute phase and predict these symptomatic hemorrhages to hold clinical utility.

The results of this study should be interpreted with caution because there are currently no methods to prove that certain EV populations in human plasma are truly of CNS origin. We presume the EV populations to be of CNS origin because the sandwich immunoassay technique will only measure L1CAM^+^/CD9^+^ double positive ELISA signals as NDEs. Free floating L1CAM or CD9^+^ EV’s lacking L1CAM would not be detected with this assay. Similarly, only EAAT1^+^/CD9^+^ and MOG^+^/CD9^+^ double positive signals would be measured as ADEs and ODEs respectively. L1CAM expression can be increased in certain tumors such as renal cell carcinoma^34^. While we excluded participants with a history of cancer, we cannot exclude the possibility that L1CAM^+^ EVs came from a tissue source outside the CNS.

Further indirect evidence supports a CNS origin for the presumed BDEs. A prior study used control plasma samples applied to anti-L1CAM (neuronal marker) and anti-CD81 (common EV marker^35^) immobilized ELISA wells. Subsequently the ratio of various other anti-neuronal and anti-non-neuronal antibodies were used to compare the two well types^21^. The ratio of these antibodies in anti-L1CAM versus anti-CD81 wells was high for neuronal markers (S100B, AchE, NRGN, CTSD, SV2A, SYNPO, SNAP25) and low for non-neuronal markers (CD9, GAPDH, ALDO, SFTPB, PSEN, BACE). This previous study also showed an increase in double labeled L1CAM^+^/CD9^+^ NDEs after cardiac surgery with timing that parallels the expected opening and closure of the BBB. In a separate prior study, EAAT1^+^ EVs were also positive for glial fibrillary astrocytic protein (GFAP) and MOG^+^ EVs were positive for myelin basic protein (MBP), lending further support to their probable astrocytic and oligodendrocytic origin^36^. Finally, data from the current study indirectly supports a probable CNS origin for BDEs based on the modest increase in BDE levels related to stroke lesion size.

This study was the first step in characterizing EVs of presumed CNS origin following ischemic stroke. We found that ADEs were preferentially increased over other brain-derived EV types in stroke patients versus controls from 5 to 30 days post-stroke. Exciting future studies await to fully investigate brain-derived EV proteins, RNAs, and lipids after stroke. This will provide much greater insight into the physiologic transition from brain injury to repair post-stroke and help determine whether EVs may be viable biomarkers for clinical events such as hemorrhagic transformation.

## Methods

### Study participants

Ischemic stroke participants and matched controls were recruited prospectively between May 29^th^, 2015 and October 28^th^, 2020 into the Biomarkers of Stroke Recovery Study (BIOREC, MedStar Georgetown University Hospital, Washington, DC). The study protocol was approved by the Georgetown University IRB (IRB # 2015-0288) and carried out according to their guidelines and regulations. All participants or their legally authorized surrogates provided written informed consent. BIOREC was a longitudinal observational study that collected blood plasma 5, 15, and 30 days after ischemic stroke in patients with mild, moderate, or severe arm motor impairment. Forty-six participants completed all assessments including a 90-day follow up and 12 participants completed assessments only through 30 days.

Control participants were recruited prospectively under the BIOREC protocol using a HIPPA waiver to screen the electronic health record (EHR) followed by full consent prior to sample collection. We used a computer algorithm to screen all patients in the MedStar EHR and identify near perfectly matched controls for the previously enrolled stroke participants based on age, race, ethnicity, sex, and cardiovascular comorbidities including hypertension, diabetes, atrial fibrillation, smoking, and statin use^22^. Only the 46 BIOREC participants who completed all study assessments had a matched control.

### Plasma collection and storage

Fasting blood samples were collected in the morning by venipuncture using EDTA tubes (Cardinal Health, OH, USA). Samples were placed on ice, delivered to the biorepository, and centrifuged at 2600 RPM for 10 min at 20 °C. Platelet-poor plasma was carefully removed with a pipette without disturbing the buffy coat and frozen at – 80 °C. All sample processing was performed within approximately 4 h from sample collection.

### Neuroimaging

All participants had a clinical MRI as part of their routine care that was assessed for lesion volume and presence and degree of hemorrhagic transformation (M.A.E.). Lesion volume was estimated using the ABC/2 method^37^. The degree of hemorrhagic transformation was graded according to the ECASS criteria adapted for MRI^38^. The 4 types of hemorrhagic transformation in order of increasing severity include: hemorrhagic infarct type 1 (HI-1, small petechial hemorrhage), hemorrhagic infarct type 2 (HI-2, confluent petechial hemorrhage), parenchymal hematoma type 1 (PH-1, hematoma < 1/3 of infarct bed), and parenchymal hematoma type 2 (PH-2, hematoma > 1/3 of infarct bed).

### Sandwich immunoassay

Mouse monoclonal antibody against human L1CAM, CD171 (Thermo Fisher Scientific, Waltham, MA), EAAT1 (Abcam, Cambridge, UK), MOG (Thermo Fisher), and CD9 (BD Biosciences, San Jose, CA) were utilized as indicators of NDEs, ADEs, ODEs and a common exosome membrane marker respectively. Control mouse IgG2a was obtained from BioLegend (San Diego, CA), and normal mouse IgG was obtained from Equitech-Bio (Kerrville, TX).

Antibodies used for immobilization (anti-CD171, anti-EAAT1, anti-MOG and control mouse IgG2a and purified normal mouse IgG) were diluted in ELISA coating buffer (BioLegend) in a final concentration of 2.5 µg/mL, and 50 µL was applied to ELISA wells (Corning #3923, Sigma Aldrich, St Louis MO). After 1-h incubation, each well was washed once with phosphate buffered saline (PBS, Thermo Fisher), and incubated with undiluted blocker casein (Thermo Fisher) for another 1 h. After each well was washed twice with PBS, ELISA wells were stored in a refrigerator.

The protocol for the double label sandwich ELISA immunoassay in this study is similar to a previous publication^21^ which demonstrated the size (100–200 nm, consistent with small EVs) and electron micrographic characterization of NDEs using this method. In the current study, EV data represent the double label of the CD171 NDEs or EAAT1 ADEs or MOG ODEs with the CD9 common EV marker. In brief, 40 µL samples of plasma were applied to ELISA wells and incubated for 1 h at room temperature. After the first washing step, each well was reacted with the CD9 biotinylated probes (50 ng/mL) supplemented with 0.8% bovine serum albumin (BSA, Thermo Fisher), 40 µg/mL mouse IgG (Equitech-Bio) for another 1 h. Biotinylation was carried out by EZ link Sulfo-NHS-LC-Biotin (Thermo Fisher) followed by the spin column procedure to remove free biotin. After the second washing step, each well was reacted with a 1/4,000 dilution of poly-horseradish peroxidase (HRP)-conjugated streptavidin (Thermo Fisher) supplemented with 10% BSA (Equitech-Bio) and 30% blocker casein (Thermo Fisher), and incubation was continued for 20 min. After the third washing step, each well was incubated for 5 min with 0.0006% H_2_O_2_ (CVS pharmacy, Irvine, CA) diluted in PBS:water (1:1) solution to remove non-specifically bound HRP conjugates. After aspiration of H_2_O_2_, each well was mixed with 1/3 dilution of chemiluminescent substrate (Super Signal, Thermo Fisher) for 4 min, then relative light units (RLU) were determined by a luminometer (Active GLO, ANSH Labs, Webster, TX). Using a standard plasma, arbitrarily assigned to 100 units/mL (U/mL). ELISA readings of RLU (Relative Light Units) were converted to U/mL by a 4-parameter logistic formula.

### Statistical analysis

Two-tailed Student’s t-tests were used to determine whether NDE, ADE, or ODE levels were significantly different between stroke and control participants, and between participants with and without hemorrhagic transformation at all time points studied. Single factor ANOVAs were performed with TOAST classification as the independent variable and BDE levels at each respective time point as the dependent variable. Stroke of other determined etiology was excluded from ANOVA analysis due to only 3 participants in this category. Chi-squared test was performed using SPSS version 28.

### Supplementary Information

Supplementary Figures.

## Data Availability

The datasets used and/or analyzed during the current study available from the corresponding author on reasonable request.
