# Supplementary material for: Elevation of astrocyte-derived extracellular vesicles over the first month post-stroke in humans
Source: Sci Rep. 2024 Mar 4;14:5272. doi: 10.1038/s41598-024-55983-w (PMC10912590; doi:10.1038/s41598-024-55983-w)
Supplement: Supplementary file 1 — Supplementary Figures. [file 41598_2024_55983_MOESM1_ESM.pdf]

Supplementary Data

Elevation of astrocyte-derived extracellular vesicles over the first month post-stroke in humans

Matthew A. Edwardson, Masato Mitsuhashi, Dennis Van Epps

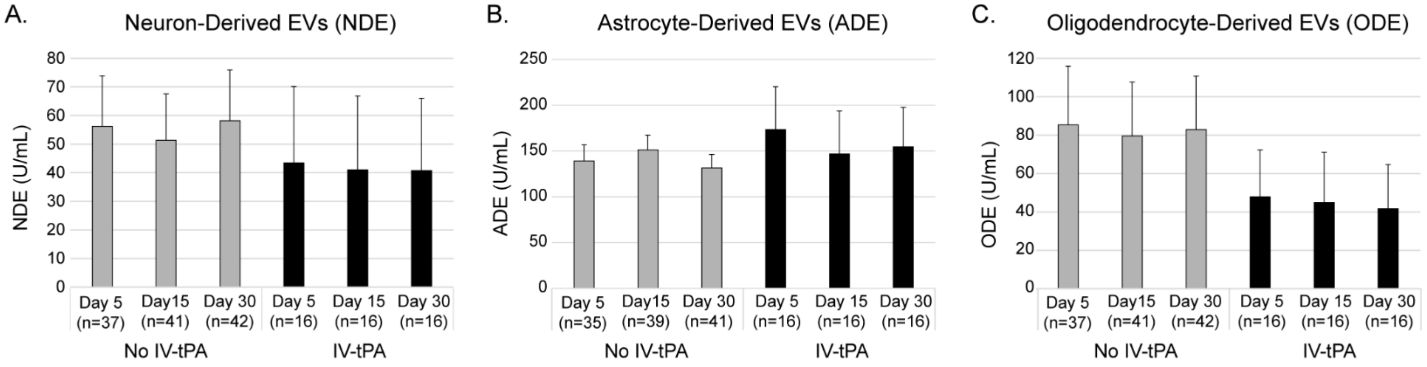

Supp Fig 1. Intravenous tPA status for ischemic stroke participants and their corresponding extracellular vesicle (EV) concentrations (mean ± SE) at 5, 15, and 30 days post-stroke for (a) neuron-derived, (b) astrocyte-derived, and (c) oligodendrocyte-derived EVs. Wherever n < 42 for no IV-tPA there were samples that returned no measurable result.

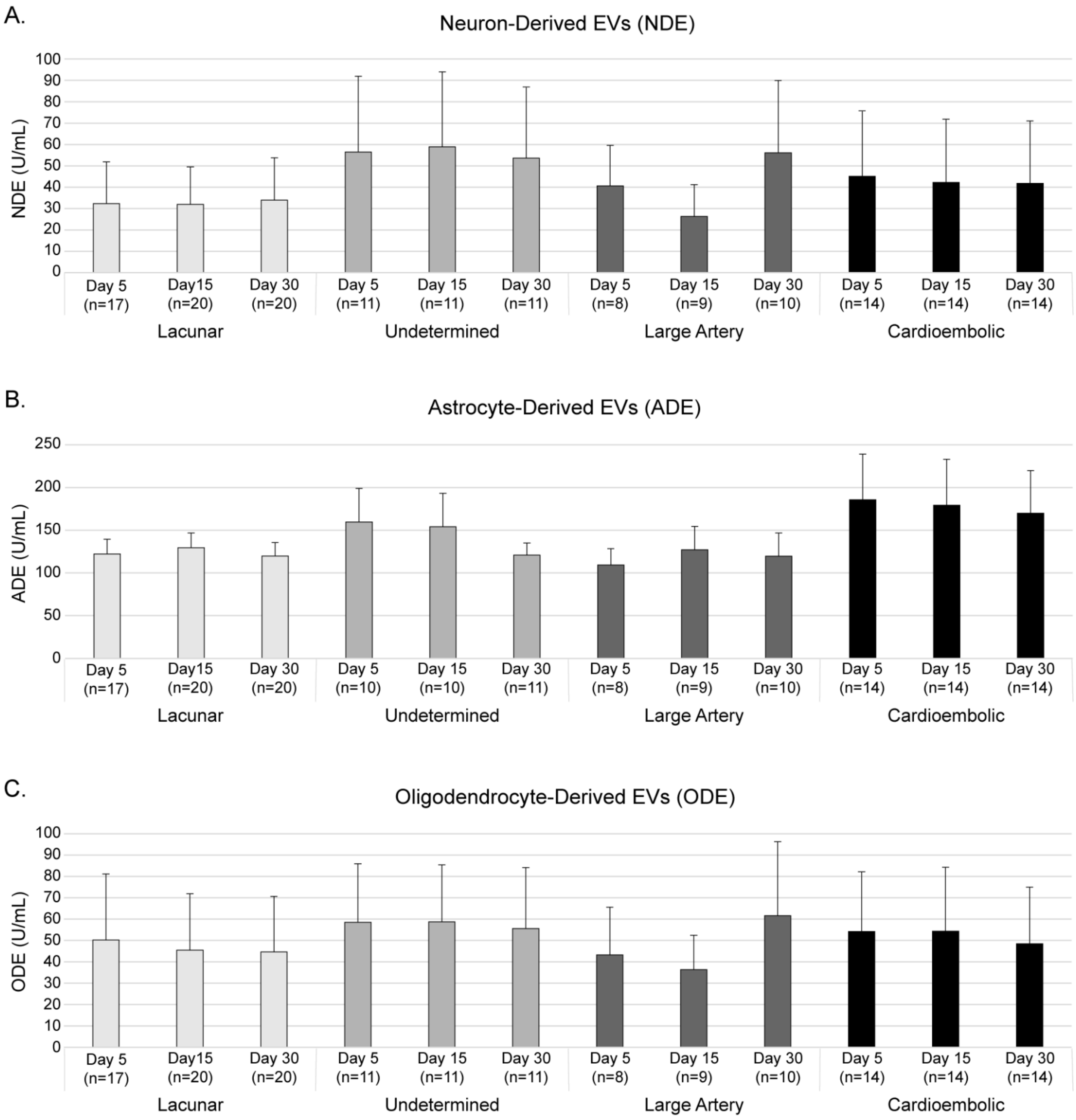

Supp Fig 2. TOAST classification categorizing the ischemic stroke etiology for participants and their corresponding extracellular vesicle (EV) concentrations (mean ± SE) 5, 15, and 30 days post-stroke for (a) neuron-derived, (b) astrocyte-derived, and (c) oligodendrocyte-derived EVs. Wherever n < 20 for lacunar, n < 11 for undetermined, or n < 10 for large artery there were samples that returned no measurable result. Undetermined = stroke of undetermined etiology. Only 3 participants had stroke of other determined etiology, so these were excluded.
